# A potential mode of action for Anakinra in patients with arthrofibrosis following total knee arthroplasty

**DOI:** 10.1038/srep16466

**Published:** 2015-11-10

**Authors:** David Dixon, Jonathon Coates, Alicia del Carpio Pons, Joanna Horabin, Andrew Walker, Nicole Abdul, Nicholas S. Kalson, Nigel T. Brewster, David J. Weir, David J. Deehan, Derek A. Mann, Lee A. Borthwick

**Affiliations:** 1 Fibrosis Research Group, Institute of Cellular Medicine, Newcastle University, Newcastle upon Tyne, NE2 4HH, UK; 2 Musculoskeletal Unit, Freeman Hospital, Newcastle Hospitals, NHS Trust, High Heaton, Newcastle upon Tyne NE7 7DN, UK

## Abstract

Arthrofibrosis is a fibroproliferative disease characterised by excessive deposition of extracellular matrix components intra-articularly leading to pain and restricted range of movement. Although frequently observed following total knee arthroplasty (TKA) no therapeutic options exist. A pilot study demonstrated that intra-articular injection of Anakinra, an IL-1R antagonist, improved range of movement and pain in patients with arthrofibrosis however the mechanism of action is unknown. We hypothesise that IL-1α/β will drive an inflammatory phenotype in fibroblasts isolated from the knee, therefore identifying a potential mechanism of action for Anakinra in arthrofibrosis following TKA. Fibroblasts isolated from synovial membranes and infra-patellar fat pad of patients undergoing TKA express high levels of IL-1R1. Stimulation with IL-1α/β induced a pro-inflammatory phenotype characterised by increased secretion of GMCSF, IL-6 and IL-8. No significant difference in the inflammatory response was observed between fibroblasts isolated from synovial membrane or infra-patellar fat pad. IL-1α/β treatments induced a pro-inflammatory phenotype in fibroblasts from both synovial membrane and infra-patellar fat pad and therefore Anakinra can likely have an inhibitory effect on fibroblasts present in both tissues *in vivo.* It is also likely that fibroblast responses in the tissues are controlled by IL-1α/β availability and not their ability to respond to it.

Total knee arthroplasty (TKA) is one of the most successful orthopaedic procedures that substantially reduces pain associated with degenerative knee joint diseases. There were 153,133 primary knee arthroplasties performed in 396 centres throughout England and Wales in a 2-year period and over 1.5 million are performed worldwide each year^1^. The surgery involves the resection of diseased or damaged bone from intra-articular areas of the knee followed by implantation of metal and polyethylene prosthetic replacements^2^. Although a very high success rate is observed with TKA surgery some 3-10% of individuals go on develop fibrosis in the form of arthrofibrosis post-surgery^3^,^4^,^5^.

Arthrofibrosis following TKA is characterised by the excessive deposition of extracellular matrix components (ECM) intra-articularly resulting in the formation of dense fibrous tissue and tissue metaplasia. This abnormal scarring of the joint prevents normal range of motion, represents a significant clinical challenge and drastically reduces quality of life of those affected individuals^4^. Currently there are no prophylactic intervention available and treatment for arthrofibrosis is restricted to aggressive physiotherapy or revision surgery^6^,^7^. However a pilot clinical study by Brown *et al.* demonstrated that intra-articular injection of Anakinra, an interleukin-1 receptor (IL-1R) antagonist, into patients who developed arthrofibrosis following TKA improvement range of motion and pain with 75% of patients able to return to prior activity levels^8^. This study is the first to identify a beneficial effect for inhibiting IL-1R1 in patients with arthrofibrosis however the mechanism of action of Anakinra in the knee is unknown.

Fibroblasts and myo-fibroblasts are cells of distinct morphology and are the most abundant cell found in connective tissue. During physiological wound healing the fibroblast plays a critical role in the restoration of tissue architecture and function however they have also been identified as one of the major effector cells involved in pathological wound repair and the development of fibrosis in multiple organs^9^,^10^,^11^. The role of the fibroblast as the major fibrogenic cell is well characterised, however less is known about the potential for these cells to contribute to inflammatory responses. Recently published work from our laboratory has shown that human lung fibroblasts express high levels of IL-1R1 and are induced to adopt a highly inflammatory phenotype in response to ligation of IL-1R1. Furthermore, mice lacking IL-1R1 are protected from bleomycin induced lung fibrosis^12^ further highlighting the importance of IL-1R1 in the development of fibrosis.

Unterhauser *et al.* have reported a 10 fold increase in the frequency of α-SMA containing contractile fibroblastic cells in knee arthrofibrotic tissue after anterior crucial ligament reconstruction^13^ suggesting a potentially important role for these cells in the pathophysiology of arthrofibrosis. In this study we demonstrate that fibroblasts isolated from the infrapatellar fat pad and synovial membrane express high levels of IL-1R1 on their cell surface and adopt a highly inflammatory phenotype in response to stimulation with IL-1α or IL-1β. We hypothesise the blockade of IL-1R1 on fibroblasts as a potential mode of action for Anakinra in patients with arthrofibrosis.

## Results

### Phenotyping of cells isolated from the infra-patellar fat pad and synovial membrane

**Figure 1:**
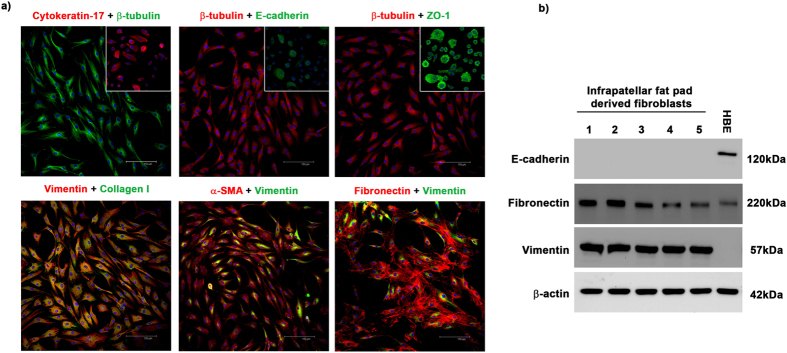
Cells isolated from the infra-patellar fat pad demonstrate a mesenchymal phenotype. (**a**) Cells isolated from the infra-patellar fat pad were cultured *in vitro* and the expression of epithelial and mesenchymal markers investigated by immunocytochemistry. Cells had little to no expression of the epithelial markers cytokeratin 17, E-cadherin and ZO-1. Insets show positive staining on human lung epithelial cells. β-tubulin was used to demonstrate cell morphology. In contrast cells expressed very high levels of the mesenchymal markers vimentin, collagen 1, α-SMA and fibronectin. DAPI was used as a nuclear counter stain. Images were acquired on a Leica TCS SP2 UV confocal microscope at x20 magnification. (**b**) Whole cell lysates of infra-patellar fat pad derived fibroblasts (n = 5) were investigated for the expression of epithelial and mesenchymal markers by Western blotting. Cells express high levels of fibronectin and vimentin but no E-cadherin. β-actin was used as a loading control. Human bronchial epithelial cells (16HBE14o- (HBE)) were used as a positive control for epithelial marker expression.

Cells were isolated from the infra-patellar fat pad and synovial membrane of patients undergoing primary TKA as described in the materials and methods. Cell phenotype was determined by assessing the expression of endothelial, epithelial and mesenchymal markers using immunocytochemistry and Western blotting. Primary human lung epithelial cells were stained as a positive control for epithelial markers. Cells from the infra-patellar fat pad (Fig. 1a, supplementary Figure 1) and synovial membrane (Supplementary Figure 2a, c) have little to no expression of the epithelial markers cytokeratin 17, E-cadherin and ZO-1 or the endothelial markers CD31 (data not shown). Inserts show positive staining for epithelial markers in human lung epithelial cells. The lack of expression of an epithelial marker, E-cadherin, was confirmed by Western blotting (Fig. 1b, Supplementary Figure 2b). β-tubulin staining visibly demonstrates the bipolar morphology of the cells, a typical characteristic of mesenchymal cells. In addition, high levels of expression of the mesenchymal makers vimentin, collagen 1, α-SMA and fibronectin were observed in cells isolated from the infra-patellar fat pad and synovial membrane. Yellow regions indicate overlapping of positive mesenchymal signals. The expression of mesenchymal markers, fibronectin and vimentin, was confirmed in n = 5 sets of patient cells by Western blotting (Fig. 1b, Supplementary Figure 2b). These data confirm that the cells isolated from the infra-patellar fat pad and synovial membrane represent a uniform population of fibroblasts/mesenchymal cells.

**Figure 2:**
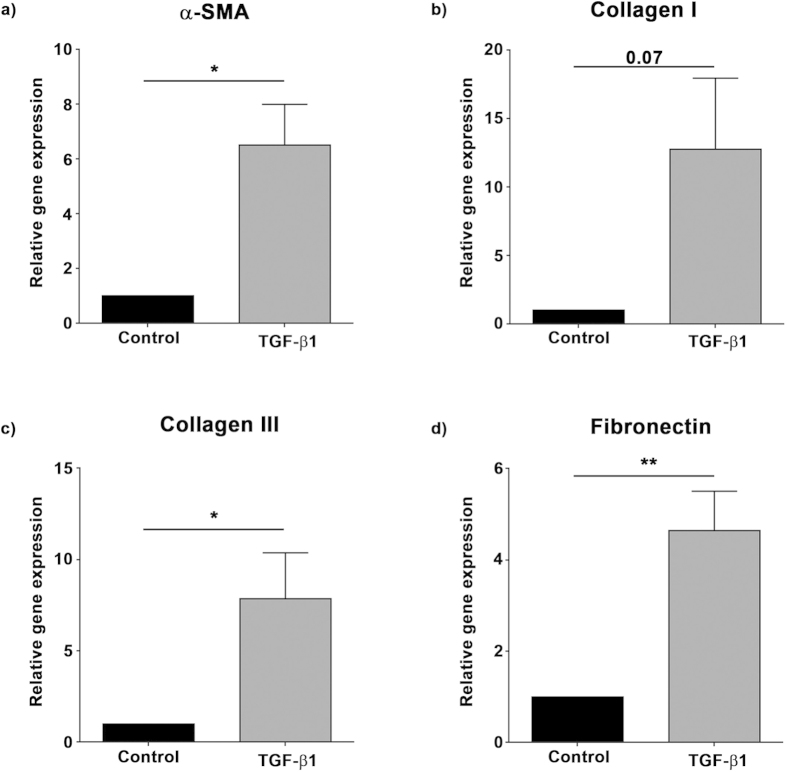
TGF-β1 treatment of infra-patellar fat pad derived fibroblasts upregulates fibrotic gene expression. Fibroblasts isolated from the infra-patellar fat pad (n = 6) were cultured *in vitro* in the presence or absence of TGF-β1 (3 ng/ml) for 48 hours and the relative gene expression of (**a**) α-smooth muscle actin (α-SMA), (**b**) collagen I, (**c**) collagen III and (**d**) fibronectin quantified by qRT-PCR. The expression of all fibrotic genes was increased by TGF-β1. Data is presented as mean ± standard error of the mean with statistical significance indicated by; *p < 0.05 and **p < 0.01.

TGF-β1 is widely accepted as a key mediator of fibrosis in multiple organs and has been demonstrated to be elevated in arthrofibrotic tissue^14^. Treatment of infra-patellar fat pad (Fig. 2) and synovial membrane (supplementary Figure 3) derived fibroblasts with TGF-β1 increased expression of α-SMA, a marker of fibroblast activation, as well as collagen I, collagen III and fibronectin, key extra cellular matrix proteins involved in fibrosis.

### Determining receptor expression on fibroblasts

**Figure 3:**
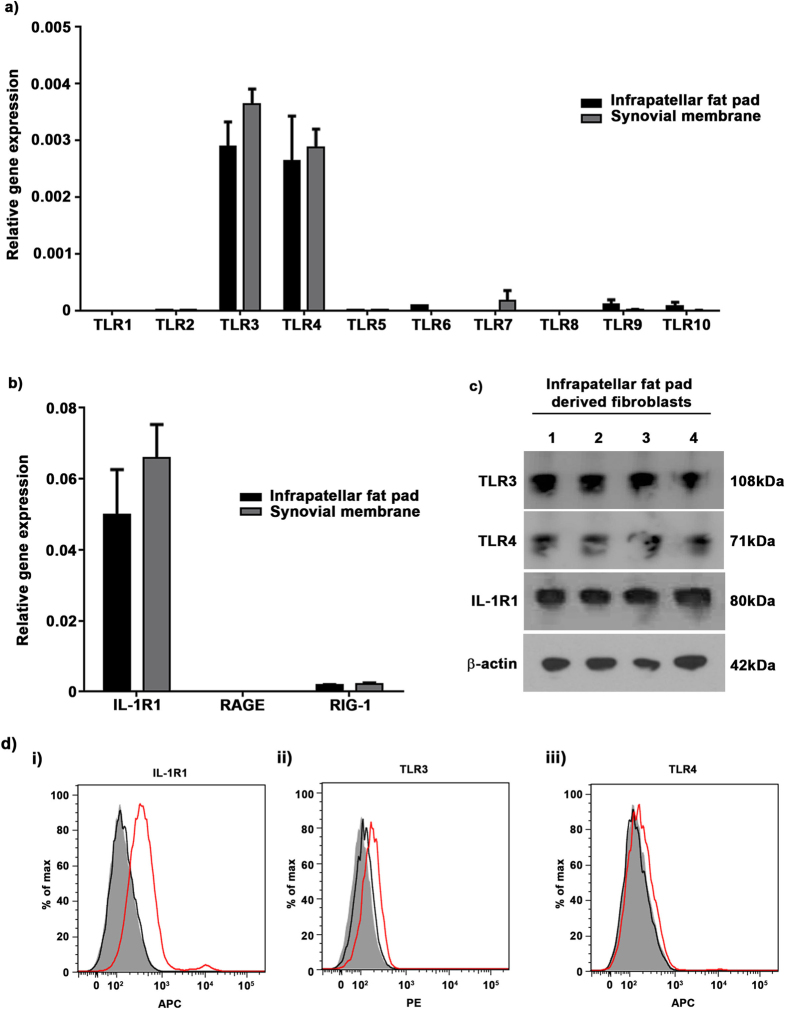
Interleukin-1 receptor 1 is highly expressed on infra-patellar pad derived fibroblasts. Fibroblasts isolated from the infra-patellar fat pad and synovial membrane (both n = 5) were cultured *in vitro* and the relative gene expression of TLR1-10, RIG-1, RAGE and IL-1R1 quantified by qRT-PCR. (**a**) TLR expression was restricted mainly to TLR3 and TLR4 with little or no expression of other TLRs. (**b**) There was no detectable expression of RAGE but RIG-1 was expressed at levels comparable to TLR3 and TLR4. In contrast IL-1R1 expression was 100–150 fold greater than other receptors. Results are normalised to GAPDH as a loading control. Data is presented as mean ± standard error of the mean. Fibroblasts isolated from the infra-patellar fat pad were investigated for the expression of TLR3, TLR4 and IL-1R1 protein by Western Blotting (n = 4) (**c**) and flow cytometry (n = 6) (**d**). Cells expressed TLR3 and TLR4 at comparable levels at the cell surface. In contrast, expression of IL-1R1 was significantly higher than other receptors (red traces). β-actin was used as a loading control **(c)**. Unstained cells (filled traces) and IgG controls (black traces) were used as flow cytometry controls **(d)**.

Previous work in the laboratory has identified that fibroblasts isolated from human lung tissue express a range of TLR’s and PRR’s that are distinct from immune cells^12^. Therefore we proceeded to investigate the expression of TLR’s (1–10) and PRR’s (IL-1R1, RAGE, RIG-1) on fibroblasts isolated from the infra-patellar fat pad and synovial membrane using qRT-PCR (Fig. 3a,b). Fibroblasts from both the infra-patellar fat pad and synovial membrane (n = 5) strongly expressed TLR 3 and TLR4 whereas all other TLRs were negligible or undetectable. The level of expression of TLR 3 and TLR4 was comparable between cells isolated from the infra-patellar fat pad and synovial membrane. IL-1R1 was expressed at very high levels, ~100 fold greater than TLR 3 and TLR4 in cells isolated from the infra-patellar fat pad and ~150 fold greater than TLR 3 and TLR4 in cells isolated from the synovial membrane. In contrast, RAGE was absent in both sets of fibroblasts and RIG-1 was present at levels comparable to TLR3 and TLR4.

We proceeded to investigate the expression of TLR3, TLR4 and IL-1R1 at a protein level using Western blotting and to investigate if these receptors are present on the cell surface using flow cytometry. The expression of TLR3, TLR4 and IL-1R1 at a protein level was confirmed in n = 4 sets of patient cells by Western blotting (Fig. 3c, Supplementary Figure 4a). Furthermore, flow cytometry demonstrated that fibroblasts from both the infra-patellar fat pad and synovial membrane (n = 6) strongly expressed IL-1R1 at the cell surface with a more limited expression of TLR3 and TLR 4 at the cell surface (Fig. 3d, Supplementary Figure 4b–d).

These data confirm that fibroblasts isolated from the infra-patellar fat pad and synovial membrane express IL-1R1 (+++), TLR3 (+) and TLR4 (+) on their cell surface.

### Stimulation of fibroblasts from the infra-patellar fat pad and synovial membrane

**Figure 4:**
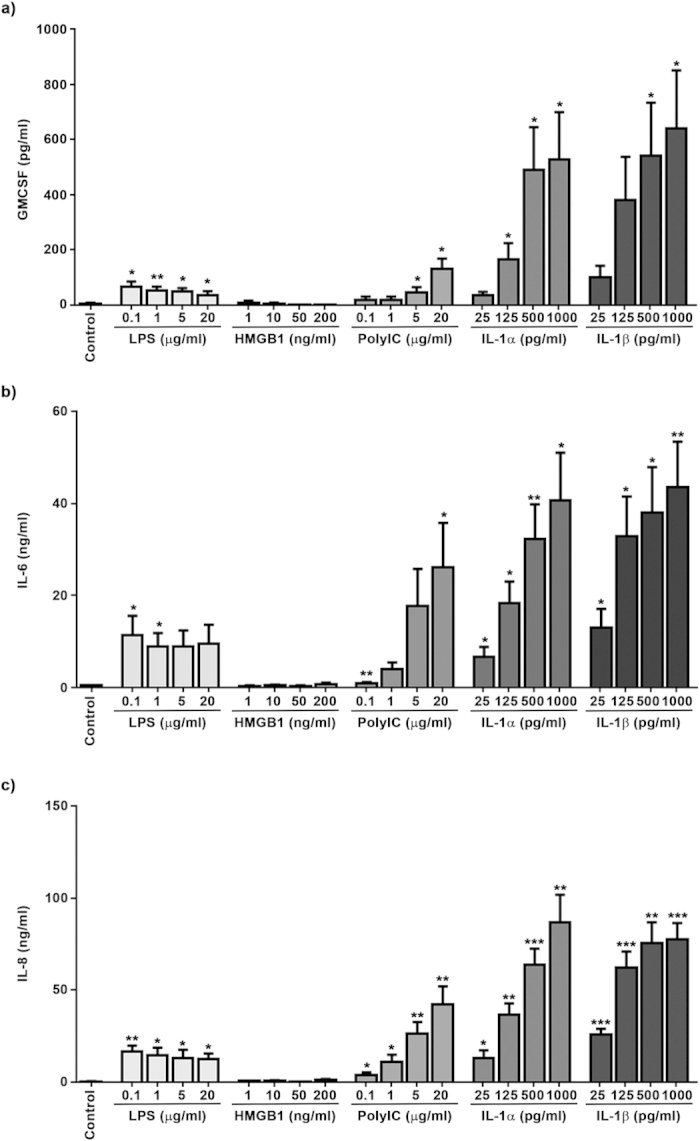
Stimulation of infra-patellar fat pad derived fibroblasts with IL-1α and IL-1β increases secretion of inflammatory mediators. Fibroblasts isolated from infra-patellar fat pad (n = 5) were stimulated with increasing doses of LPS (0.1–20 μg/ml), HMGB1 (1–200 ng/ml), PolyIC (0.1–20 μg/ml), IL-1α and IL-1β (both 25–1000 pg/ml) for 24 hours and the secreted levels of GM-CSF (**a**), IL-6 (**b**) and IL-8 (**c**) determined by ELISA. All three molecules demonstrated a dose dependant increase in secretion in response to PolyIC, IL-1α and IL-1β. LPS induced maximum release of all three molecules at the lowest concentration used (0.1 μg/ml) with higher concentrations having no additional effect. The cells showed no significant response to HMGB-1. Data is presented as mean ± standard error of the mean with statistical significance compared to control indicated by; *p < 0.05, **p < 0.01 and ***p < 0.001.

Fibroblasts isolated from infra-patellar fat pad and synovial membrane (n = 6) were stimulated for 24 hours with increasing doses of receptor ligands (LPS—TLR4, HMGB1—RAGE, PolyIC—TLR3, IL-1α & IL-1β—both IL-1R1) and the secretion of pro-inflammatory cytokines (IL-6, IL-8, and GM-CSF) investigated. Treatment of fibroblasts from both the infra-patellar fat pad and synovial membrane with LPS at the lowest dose tested in this study (0.1 mg/ml) maximized secretion of IL-6, IL-8 and GM-CSF, with higher doses (up to 20 ng/ml) exhibiting a reduced response. As expected given the lack of RAGE expression in these cells, HMGB-1 up to concentrations of 200 ng/ml had little to no effect on the secretion of IL-6, IL-8 and GM-CSF in fibroblasts from either the infra-patellar fat pad or the synovial membrane. In contrast, treatment with PolyIC induced a dose dependant increase of IL-6, IL-8 and GM-CSF secretion in fibroblasts isolated from both tissues. The magnitude of the GM-CSF response to PolyIC was similar to that induced by LPS but PolyIC induced significantly higher levels (~2–3 fold) of IL-6 and IL-8. Finally, treatment with IL-1α and IL-1β induced a dose dependant increase in the secretion of IL-6, IL-8 and GMCSF in fibroblasts isolated from both tissues. The magnitude of the response to IL-1α and IL-1β was significantly higher for IL-6, IL-8 and GMCSF compared to PolyIC and LPS (Fig. 4, Supplementary Figure 5). No secretion of TNFα from fibroblasts isolated from the infra-patellar fat pad or synovial membrane was detected in response to any of the treatment (data not shown).

**Figure 5:**
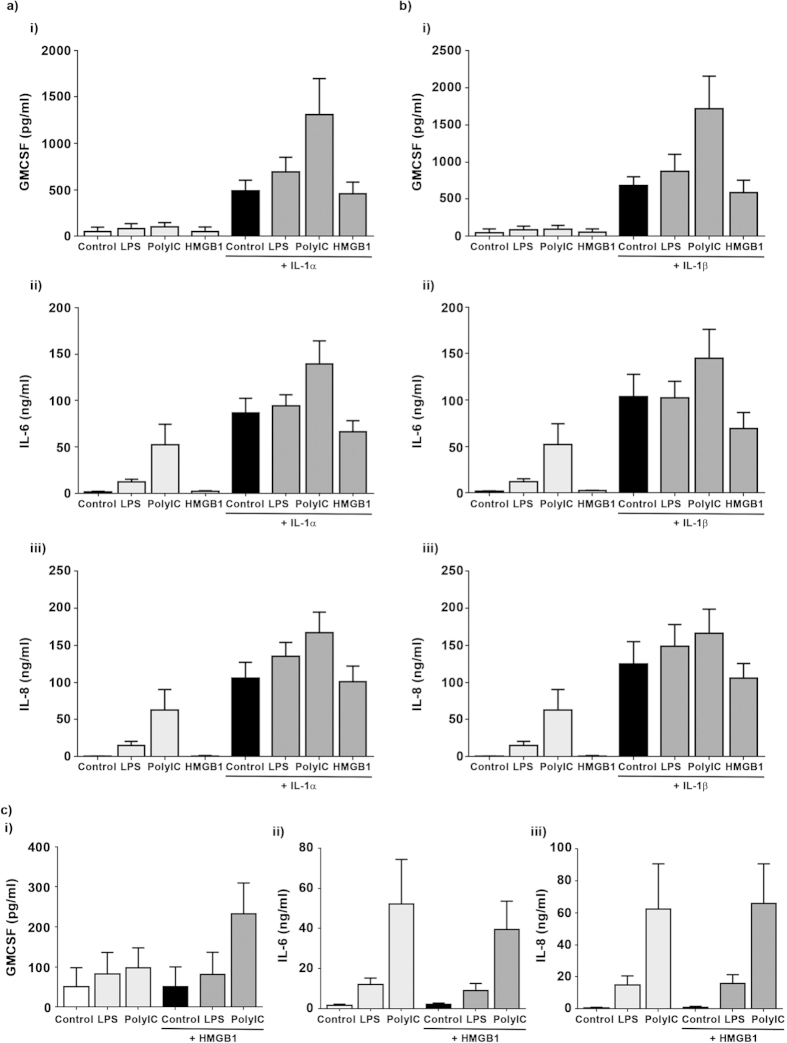
PolyIC and LPS increase IL-1α/IL-1β induced secretion of inflammatory mediators in infra-patellar fat pad derived fibroblasts. Fibroblasts isolated from infra-patellar fat pads (n = 5) were stimulated with IL-1α, IL-1β (both 500 pg/ml), LPS, PolyIC (both 5 μg/ml) or HMGB-1 (50 ng/ml) alone or in combination for 24 hours and the secretion of IL-8, IL-6 and GM-CSF quantified by ELISA. (**a**,**b**) IL-1α and IL-1β induced secretion of GMCSF was accentuated by PolyIC. In contrast, Poly IC induced only an additive effect on IL-1α and IL-1β induced secretion of IL-6 and IL-8. LPS induced an additive effect on IL-1α and IL-1β induced secretion of GMCSF, IL-6 and IL-8 while HMGB1 had no effect. (**c**) Co-treatment with HMGB-1 and LPS or PolyIC was not significantly different to treatment with LPS or PolyIC alone. Data presented as mean ± standard error of the mean.

Given the varied inflammatory response induced by single ligand stimulation, we proceeded to determine if co-stimulation with ligands would produce additive, synergistic or inhibitive responses. To do this we stimulated synovial membrane and infra-patellar fat pad derived fibroblasts (n = 6) with IL-1α (500 pg/ml), IL-1β (500 pg/ml), LPS (5 μg/ml), PolyIC (5 μg/ml) or HMGB-1 (50 ng/ml) alone or in combination for 24 hours and measured secreted levels of IL-8, IL-6 and GM-CSF by ELISA. As we previously demonstrated, IL-1α and IL-1β treatment induced the secretion of GMCSF, IL-6 and IL-8 from fibroblasts isolated from the infra-patellar fat pad and synovial membrane. Co-stimulation with IL-1α or IL-1β and PolyIC induced a synergistic response to greatly increase GMCSF secretion. In contrast an additive effect on IL-6 and IL-8 secretion was observed by co-stimulating with IL-1α or IL-1β and PolyIC. Co-stimulation with IL-1α or IL-1β and LPS induced an additive effect on GMCSF, IL-6 and IL-8 secretion in fibroblasts isolated from both tissues. Co-stimulation with IL-1α or IL-1β and HMGB1 had no additional effect on GMCSF, IL-6 and IL-8 secretion compared to treatment with IL-1α or IL-1β alone. Treatment of fibroblasts with HMGB-1 and polyIC or LPS had little to no effect on GMCSF, IL-6 and IL-8 secretion compared to treatment with polyIC or LPS alone (Fig. 5, Supplementary Figure 6).

IL-1α and IL-1β induce a potent pro-inflammatory phenotype in fibroblasts isolated from both tissues. PolyIC and LPS similarly induce a pro-inflammatory phenotype but the magnitude of the response is reduced. PolyIC has a strong synergistic effect with IL-1α and IL-1β on GM-CSF secretion from fibroblasts isolated from both tissues.

## Discussion

TKA alleviates pain and restores function in patients who have degenerative knee joint diseases. Although a highly successful operation, some 3–10% of individuals go on develop fibrosis in the form of arthrofibrosis post-surgery. Arthrofibrosis following TKA is described as a fibrosing pathology of the synovial membrane^15^ and infra-patellar fat pad^16^ for which there is no recommended therapeutic intervention. However, a pilot clinical study by Brown *et al.*^8^ demonstrated a marked improvement in range of motion and pain in patients with arthrofibrosis suggesting an important role for IL-1R1 in the pathology. In this study we have shown that fibroblasts isolated from the infra-patellar fat pad and synovial membrane express high levels of IL-1R1 on the cell surface and are induced to adopt a highly inflammatory phenotype in response to stimulation with IL-1α and IL-1β. In contrast, TGF-β1 induced an increase in fibrotic gene expression suggesting that these cells are plastic and can modulate their phenotype in response to changes in the microenvironment.

Our data is in agreement with previous studies in other tissues showing that IL-1α and IL-1β can drive an inflammatory phenotype in fibroblasts. For example a study by Boxman *et al.* has demonstrated that IL-1α released from activated keratinocytes induces an increase in IL-8 and IL-6 production in human dermal fibroblasts^17^. IL-1α has been shown to be a potent stimulator of GM-CSF, IL-6 and IL-8 expression in fibroblasts isolated from histologically normal human duodenal biopsy tissues^18^. IL-1β has been demonstrated to stimulate the secretion of IL-6 and IL-8 in rheumatoid arthritis fibroblast like synoviocytes^19^. Finally, previous work from our laboratory has shown that IL-1α released from damaged human lung epithelial cells induces a potent pro-inflammatory phenotype in human lung fibroblasts^12^.

There is also a significant body of work demonstrating that IL-1α, IL-1β and IL-1R1 are involved in inflammation and fibrosis *in vivo*. For example, IL-1R1 KO mice are protected from bleomycin induced lung fibrosis and nasal installation of recombinant murine IL-1β into C57Bl6 mice induced lung tissue remodelling comparable to bleomycin^20^. Furthermore, adenoviral induced transient overexpression of IL-1β in rat lungs was accompanied by local increase in proinflammatory cytokines and severe progressive tissue fibrosis^21^. Liu *et al.* demonstrated that IL-1β plays a critical role in radiation induced skin fibrosis^22^. Stimulation of human proximal tubule cells isolated from histologically normal segments of renal cortex with IL-1β enhanced fibronectin production and promoted α-smooth muscle actin expression^23^ and IL-1R1 KO & IL-1β KO mice are protected from injury and collagen deposition in the unilateral ureteric obstruction model of kidney fibrosis^24^. Finally, IL-1R1 KO mice also exhibited ameliorated liver damage and reduced fibrogenesis in response to treatment with thioacetamide^25^. However, there are currently no studies that we are aware of that have selectively depleted IL-1R1 on fibroblasts to confirm a direct involvement of the receptor expressed on fibroblasts in fibrosis.

Our data shows that fibroblasts isolated from the synovial membrane and infrapatellar fat pad express similar levels of IL-1R1 on the cell surface and that the IL-1α/IL-1β induced inflammatory response is of similar magnitude. It is therefore likely that the induction of an inflammatory response in fibroblast *in vivo* is regulated by the availability/expression of IL-1α and IL-1β in the tissue and that Anakinra can exert an inhibitory effect on IL-1R1 expressed on fibroblasts present in both tissues *in vivo*. However further research is required to determine the *in vivo* tissue/tissues targeted by Anakinra in patients with arthrofibrosis to help improve our understanding of the pathology and how it can be treated.

In summary, there are currently no approved therapeutic interventions available for the treatment of arthrofibrosis excluding a TKA revision surgery. Revision surgeries put patients at risk of complications from anaesthesia, infection and pain amongst others. Revision surgery also comes at a great cost including surgeon’s time, theatre use, theatre staff and equipment. A pilot study has demonstrated a beneficial effect of treatment with Anakinra in patients with arthrofibrosis. In this study we propose a potential mechanism for the reported beneficial impact of Anakinra on patients with arthrofibrosis, *via* inhibition of IL-1R1 on fibroblasts.

## Methods

### Patient recruitment and ethics

This study was performed in accordance with approval from the Newcastle and North Tyneside Local Regional Ethics Committee and informed written consent from all study patients (12/NE/0395).

### Tissue processing and cell isolation

Infra-patellar fat pad and synovial membrane were acquired from patients (n = 6) undergoing primary TKA at the Freeman Hospital Newcastle upon Tyne NHS Foundation Trust. Tissues were homogenized and digested in 10 ml of supplement free Dulbecco’s Modified Eagle’s Medium (DMEM) (Sigma) containing 100 cdg units/ml of collagenase (Sigma). Digested tissue was passed through a 100 μm filter and the collagenase neutralized with complete DMEM (10% FCS, 100 μg/ml Streptomycin, 100 U/ml penicillin, 1% L-glutamate and 0.5 μg/ml amphotericin B). Cells were pelleted and re-suspended in complete DMEM before transferring into T-75 flasks. Cells were cultured at 37 °C and 5% CO_2_ until > 90% confluent. Cells were cryopreserved (DMSO freezing media, Sigma) at P0 or passaged and cryopreserved at P1. Cells were reanimated and used between P3 and P5 for experiments.

PBEC from stable lung transplant recipients were isolated as previously described^26^. Human bronchial epithelial cell line (16HBE14o^−^) were cultured as described previously^27^.

### Cell treatments

Cells were seeded at 7 × 10^3^ cells/cm^2^ and treated for 24 hours with increasing doses of recombinant human HMGB-1 (1–200 ng/ml), IL-1α (25–1000 pg/ml), IL-1β (25–1000 pg/ml) (all R&D), LPS (0.1–20 μg/ml) and PolyIC (0.1–20 μg/ml) (both Sigma) or for 24 hours with LPS (5 μg/ml), PolyIC (5 μg/ml), HMGB-1 (50 ng/ml), IL-1α (500 pg/ml) and IL-1β (500 pg/ml) alone or in combination. Cell culture supernatants were harvested for ELISA. For PCR, cells were seeded at 7 × 10^3^ cells/cm^2^ and treated for 48 hours with TGF-β1 (3 ng/ml).

### Immunocytochemistry

Cells (1 × 10^4^ cells/cm^2^) were seeded onto coverslips for 24 hours and fixed in 4% paraformaldehyde. Subsequently cells were washed and quenched with 100 mM glycine before permeabilisation using 0.1% Triton X-100 and blocking with 5% bovine serum albumin (BSA). Primary antibodies (α-SMA (Ab5694, Abcam), β-tubulin (T4026, Sigma), collagen I (C2456, Sigma), cytokine 17 (Ab53707, Abcam), E-cadherin (610181, BD Bioscience), fibronectin (F3648, Sigma), vimentin (Ab92547, Abcam & M7020, Dako) and ZO-1 (33–9100, Zymed)) were added and incubated overnight at 4° C in 5% BSA. Cells were washed with 0.2% tween-20 in PBS and then incubated with secondary antibodies (anti-rabbit TRITC (T6778, Sigma) and anti-mouse Alexafluor 488 (A11001, Molecular Probes)) for 60 mins in 5% BSA. Cover slips were washed and mounted in mounting media containing DAPI (Vectra Shield). Images acquired using a Leica TCS SP2 UV confocal microscope.

### Western Blot

Whole cell lysates were prepared by sonication of cells. Protein concentration of samples were determined by BCA protein assay (Thermo Scientific) as per manufacturer’s instructions. Samples (5 or 10 μg) were separated under denaturing conditions on a NuPage 4–12% Bis-Tris gels (Invitrogen). Gels were then transferred onto Hybond-P polyvinylidene fluoride (PVDF) membranes, blocked and incubated with primary antibody (β-actin (A2228, Sigma), E-cadherin (610181, BD Bioscience), fibronectin (F3648, Sigma), vimentin (Ab92547, Abcam), TLR3 (12–9039, eBioscience), TLR4 (51–9917–73, eBioscience) and IL-1R1 (AF269, R&D). Membranes were washed and incubated with HRP conjugated secondary antibodies (Ab6820 & Ab6721, Abcam) before being washed and visualised using SuperSignal Pico Chemiluminescent Substrate (Thermo-Scientific) and X-ray film. Full blots for all antibodies are shown in supplementary Figure 7.

### RNA Isolation

RNA was isolated using NucleoSpin**®** RNA II kit (MACHEREY-NAGEL) as per manufacturer’s instructions. cDNA was generated using the AffinityScript™ Multiple Temperature RT kit (Alignment Technologies) as per manufactures instructions.

### qRT-PCR

qRT-PCR was used to investigate gene expression of TLR 1–10, RIG, RAGE, IL-1R1, α-SMA, collagen I/III and fibronectin (Table 1). Master mix was generated by aliquoting sybr green (Sigma-Aldrich), RNase-free H_2_O and the appropriate primer to duplicate wells on a 96 well PCR plate (Applied Biosystems). cDNA samples were diluted to 5ng/ml before adding to the plate. Plates were run as per protocol (7500 Fast RT-PCR, Applied Biosystems). Cycle threshold values were averaged and relative gene expression calculated against GAPDH.

### Flow cytometry

Cells were preincubated in LIVE/DEAD (L34955, Life Technologies) to determine cell viability followed by incubation with specific mAbs (TLR3 (eBioscience 12–9039), TLR4 (eBioscience 51–9917–73) and IL-1R1 (R&D AF269)) diluted in PBS/1% BSA. After staining, cells were washed and resuspended in PBS/1% BSA for analysis. Isotype controls (IC108A, R&D & 12–4714–71, eBioscience) were included as negative controls. Data was acquired on a BD FACSCanto II and analyzed using FlowJo software.

### ELISA

IL-6, IL-8, GM-CSF levels in cell culture media was measured using ELISA DuoSet kit (R&D Systems) as per manufacturer’s protocols. Level of detection – GMCSF – 15.6 pg/ml, IL-8–31.2 pg/ml & IL-6–9.4 pg/ml.

### Statistical analysis

Results are presented as mean ± standard error of the mean (SEM). Differences between treatment groups were analysed using students t-test (Graphpad Prism 6 statistical software). Probability (P) values of <0.05 were regarded as significant.

## Additional Information

**How to cite this article**: Dixon, D. *et al.* A potential mode of action for Anakinra in patients with arthrofibrosis following total knee arthroplasty. *Sci. Rep.* **5**, 16466; doi: 10.1038/srep16466 (2015).

## Supplementary Material

Supplementary Information

## Figures and Tables

**Table 1:** List of PCR primers.

Gene Name	Forward Primer Sequence	Reverse Primer Sequence
TLR1	CCTAAAGACCTATCCCAGAA	ACAGTAGGGTGGCAAGAAAT
TLR2	TTGTGGATGGTGTGGGTCTT	AGGTCACTGTTGCTAATGTA
TLR3	TATTTCCCTTGCCTCACTCC	TGGTTAGGTTGAGTATGTGT
TLR4	TTTTTCTAATCTGACCAATC	TCATAGGGTTCAGGGACAGG
TLR5	TACCCCCTTGACTATTGACA	ATAACCATCTTTCAATACAG
TLR6	TTCCATTTTGTTTGCCTTAT	TTATGGGAAAGTCTCAAAAC
TLR7	GATTTACTCCATTCAACAGC	TGTCGTTCATCATCAGTTTC
TLR8	ATGTTCCTTCAGTCGTCAAT	TTTTGCTTTTTCTCATCACA
TLR9	TACCTTGCCTGCCTTCCTAC	TGTCACCAGCCTTTCCTTGT
TLR10	TTATGACAGCAGAGGGTGATG	GGAGTTGAAAAAGGAGGTTA
IL-1R	CCTAAAGACCTATCCCAGAA	ACAGTAGGGTGGCAAGAAAT
RAGE	TTGTGGATGGTGTGGGTCTT	AGGTCACTGTTGCTAATGTA
RIG-1R	AGAGCACTTGTGGACGCTTT	TGCAATGTCAATGCCTTCAT
α-SMA	GCTGTTTTCCCATCCATTGTG	TTGGTGAGTAGTCCATGTTCT
Collagen I	CCTGGATGCCATCAAAGTCT	AATCCATCGGTCATGCTCTC
Collagen III	GTCCATGGATGGTGGTTTTC	CACCTTCATTTGACCCCATC
Fibronectin	ACCAACCTACGGATGACTCG	GCTCATCATCTGGCCATTTT
