# Supplementary material for: A potential mode of action for Anakinra in patients with arthrofibrosis following total knee arthroplasty
Source: Sci Rep. 2015 Nov 10;5:16466. doi: 10.1038/srep16466 (PMC4639732; doi:10.1038/srep16466)
Supplement: Supplementary Information [file srep16466-s1.pdf]

A potential mode of action for Anakinra in patients with arthrofibrosis following total knee arthroplasty

David Dixon[1], Jonathon Coates, Alicia del Carpio Pons, Joanna Horabin[1], Andrew Walker[1] , Nicole Abdul[1,2], Nicholas S Kalson[1,2], Nigel T Brewster[2] , David J Weir[2], David J Deehan[1,2], Derek A Mann[1], Lee A Borthwick[1*]

Supplementary figure legends

Supplementary figure 1 – Cells isolated from the infra-patellar fat pad are negative for epithelial markers

Cells isolated from the infra-patellar fat pad were cultured *in vitro* and the expression of epithelial markers investigated by immunocytochemistry. Cells had little to no expression of the epithelial markers cytokeratin 17, E-cadherin and ZO-1. β-tubulin was used to demonstrate cell morphology and DAPI was used as a nuclear counter stain. Images were acquired on a Leica TCS SP2 UV confocal microscope at x20 magnification.

Supplementary figure 2 – Cells isolated from the synovial membrane demonstrate a mesenchymal phenotype

(a, c) Cells isolated from the synovial membrane were cultured *in vitro* and the expression of epithelial and mesenchymal markers investigated by immunocytochemistry. Cells had little to no expression of the epithelial markers cytokeratin 17, E-cadherin and ZO-1. Insets show positive staining on lung epithelial cells. β-tubulin was used to demonstrate cell morphology. In contrast cells expressed very high levels of the mesenchymal markers vimentin, collagen 1, α-SMA and fibronectin. DAPI was used as a nuclear counter stain. Images were acquired on a Leica TCS SP2 UV confocal microscope at x20 magnification.

(b) Whole cell lysates from cells isolated from the synovial membrane (n=5) were investigated for the expression of epithelial and mesenchymal markers by Western Blotting. Cells express high levels of fibronectin and vimentin but no E-cadherin. β-actin was used as a loading control. Human bronchial epithelial cells (16HBE14o-) were used as a positive control for epithelial marker expression.

Supplementary figure 3 – TGF-β1 treatment of synovial membrane derived fibroblasts upregulates fibrotic gene expression

Fibroblasts isolated from the synovial membrane (n=3) were cultured *in vitro* in the presence or absence of TGF-ß1 (3ng/ml) for 48 hours and the relative gene expression of **(a)** α-smooth muscle actin (α-SMA), **(b)** collagen I, **(c)** collagen III and **(d)** fibronectin quantified by qRT-PCR. The

expression of all fibrotic genes was increased by TGF-β1. Data is presented as mean ±standard error of the mean with statistical significance indicated by; *p<0.05 and **p<0.01.

Supplementary figure 4 – Interleukin-1 receptor 1 is highly expressed on synovial membrane derived fibroblasts

Fibroblasts isolated from the synovial membrane were investigated for the protein expression of TLR3, TLR4 and IL-1R1 by Western blotting (n=4) **(a)** and flow cytometry (n=6) **(b-d).** Cells expressed TLR3 and TLR4 at comparable levels at the cell surface. In contrast, expression of IL-1R1 was significantly higher than other receptors (red traces). β-actin was used as a loading control **(a)**. Unstained cells (filled traces) and IgG controls (black traces) were used as controls for flow cytometry **(b)**.

Supplementary figure 5 – Stimulation of synovial membrane derived fibroblasts with IL-1α and IL-1β increases secretion of inflammatory mediators.

Fibroblasts isolated from synovial membrane (n=5) were stimulated with increasing doses of LPS (0.1-20μg/ml), HMGB1 (1-200ng/ml), PolyIC (0.1-20μg/ml), IL-1α and IL-1β (both 25-1000pg/ml) for 24 hours and the secreted levels of GM-CSF **(a)**, IL-6 **(b)** and IL-8 **(c)** determined by ELISA. All three molecules demonstrated a dose dependant increase in secretion in response to PolyIC, IL-1α and IL-1β. LPS induced maximum release of all three molecules at the lowest concentration used (0.1μg/ml) with higher concentrations having no additional effect. The cells showed no significant response to HMGB-1. Data is presented as mean ±standard error of the mean with statistical significance compared to control indicated by; *p<0.05 and **p<0.01.

Supplementary figure 6 – PolyIC and LPS increase IL-1α/IL-1β induced secretion of inflammatory mediators in synovial membrane derived fibroblasts

Fibroblasts isolated from synovial membrane (n=5) were stimulated with IL-1α, IL-1β (both 500pg/ml), LPS (5μg/ml), PolyIC (5μg/ml) or HMGB-1 (50ng/ml) alone or in combination for 24 hours and the secretion of IL-8, IL-6 and GM-CSF quantified by ELISA. **(a-b)** IL-1α and IL-1β induced secretion of GMCSF was accentuated by PolyIC. In contrast, Poly IC induced only an

additive effect on IL-1α and IL-1β induced secretion of IL-6 and IL-8. LPS induced an additive effect on IL-1α and IL-1β induced secretion of GMCSF, IL-6 and IL-8 while HMGB1 had no effect. **(c)** Co-treatment with HMGB-1 and LPS or PolyIC was not significantly different to treatment with LPS or PolyIC alone. Data presented as mean ± standard error of the mean.

Supplementary figure 7 – Complete Western blots for all experiments

Full length Western blots for results shown in figure 1, figure 3, supplementary figure 2 and supplementary figure 4.

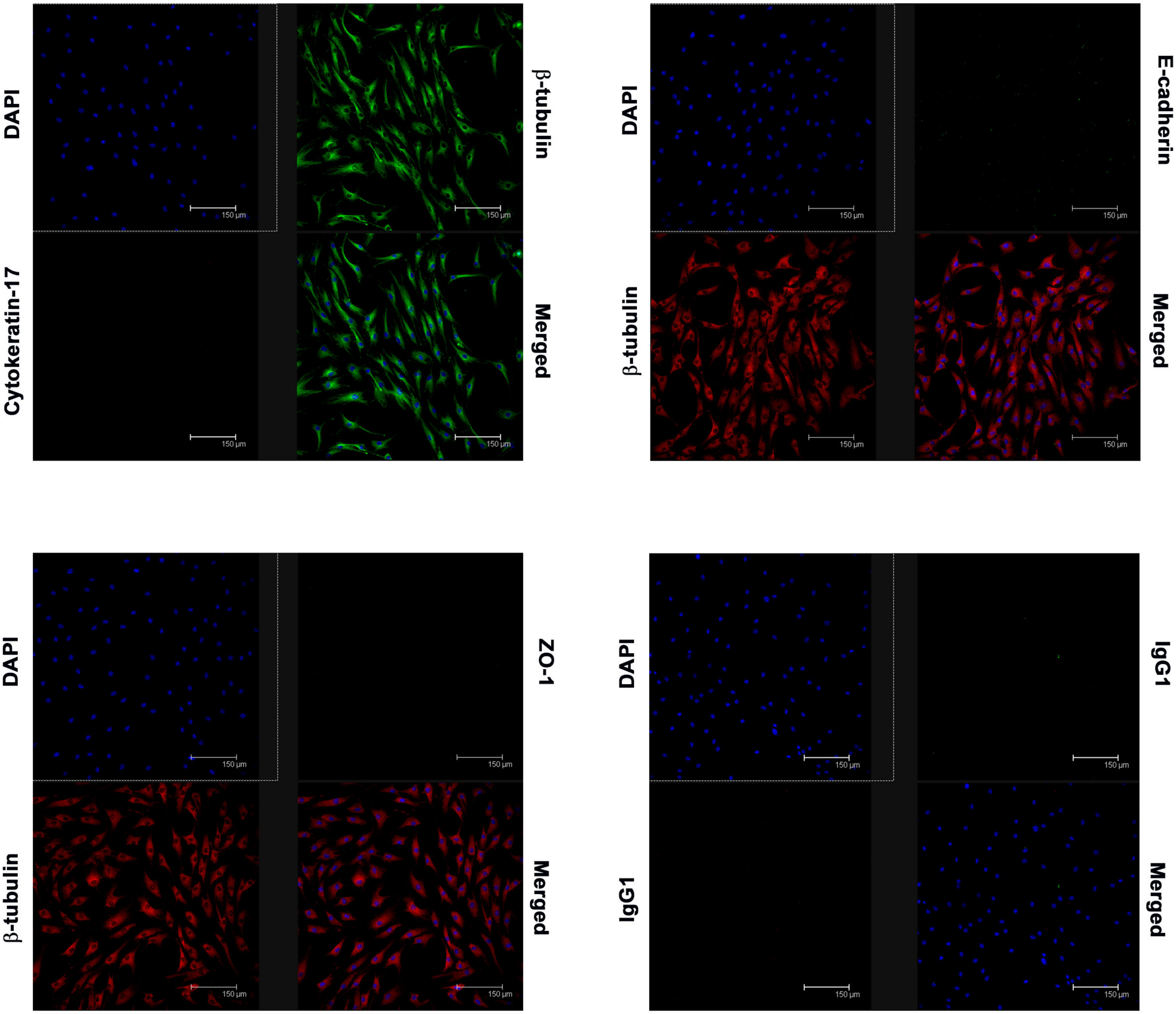
DAPI
Cytokeratin-17
β-tubulin
Merged
150 µm
DAPI
β-tubulin
E-cadherin
Merged
150 µm
DAPI
β-tubulin
ZO-1
Merged
150 µm
DAPI
IgG1
IgG1
Merged
150 µm

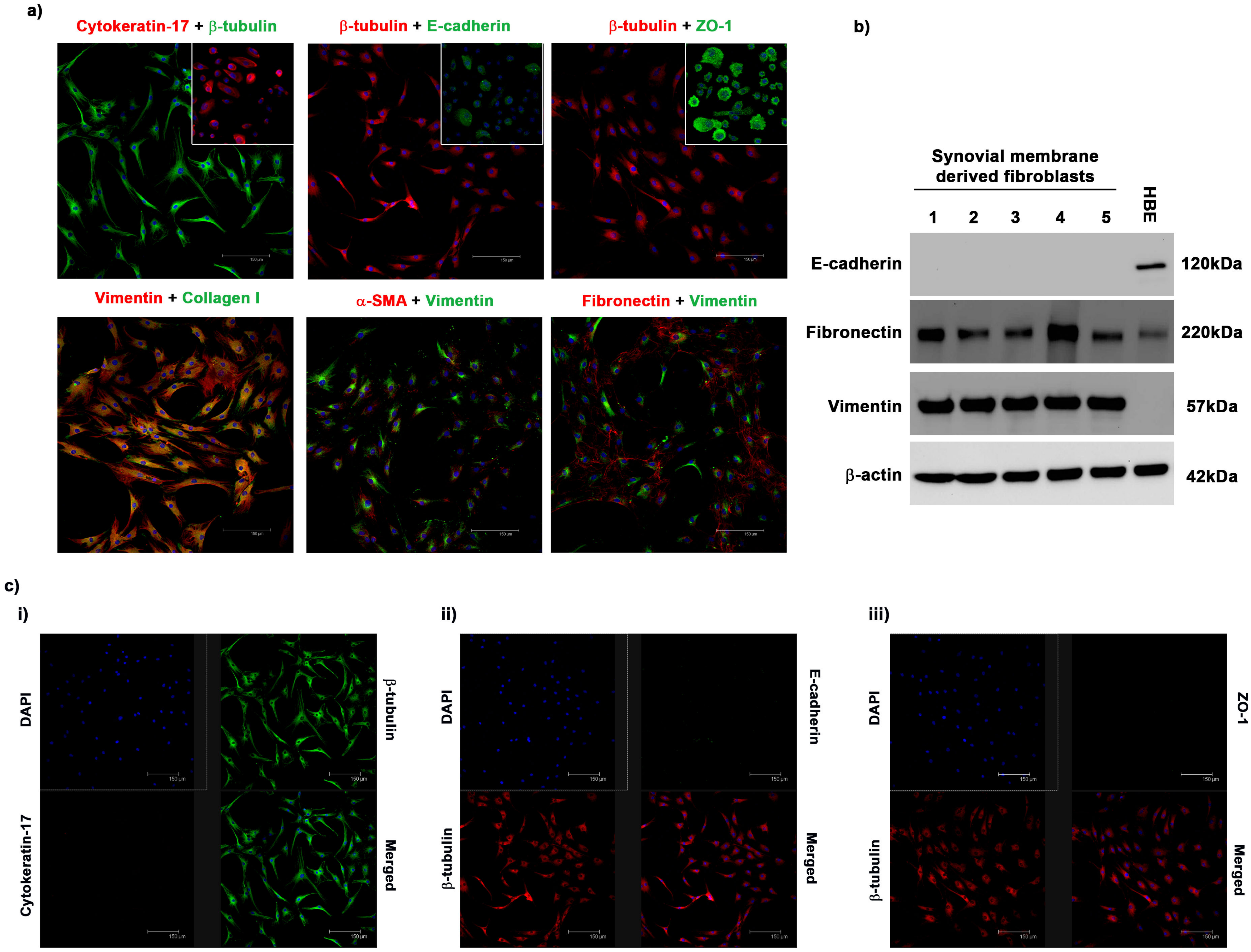
a)
Cytokeratin-17 + β-tubulin
β-tubulin + E-cadherin
β-tubulin + ZO-1
Vimentin + Collagen I
α-SMA + Vimentin
Fibronectin + Vimentin
b)
Synovial membrane derived fibroblasts
1
2
3
4
5
HBE
E-cadherin
120kDa
Fibronectin
220kDa
Vimentin
57kDa
β-actin
42kDa
c)
i)
DAPI
β-tubulin
Cytokeratin-17
Merged
ii)
DAPI
E-cadherin
β-tubulin
Merged
iii)
DAPI
ZO-1
β-tubulin
Merged

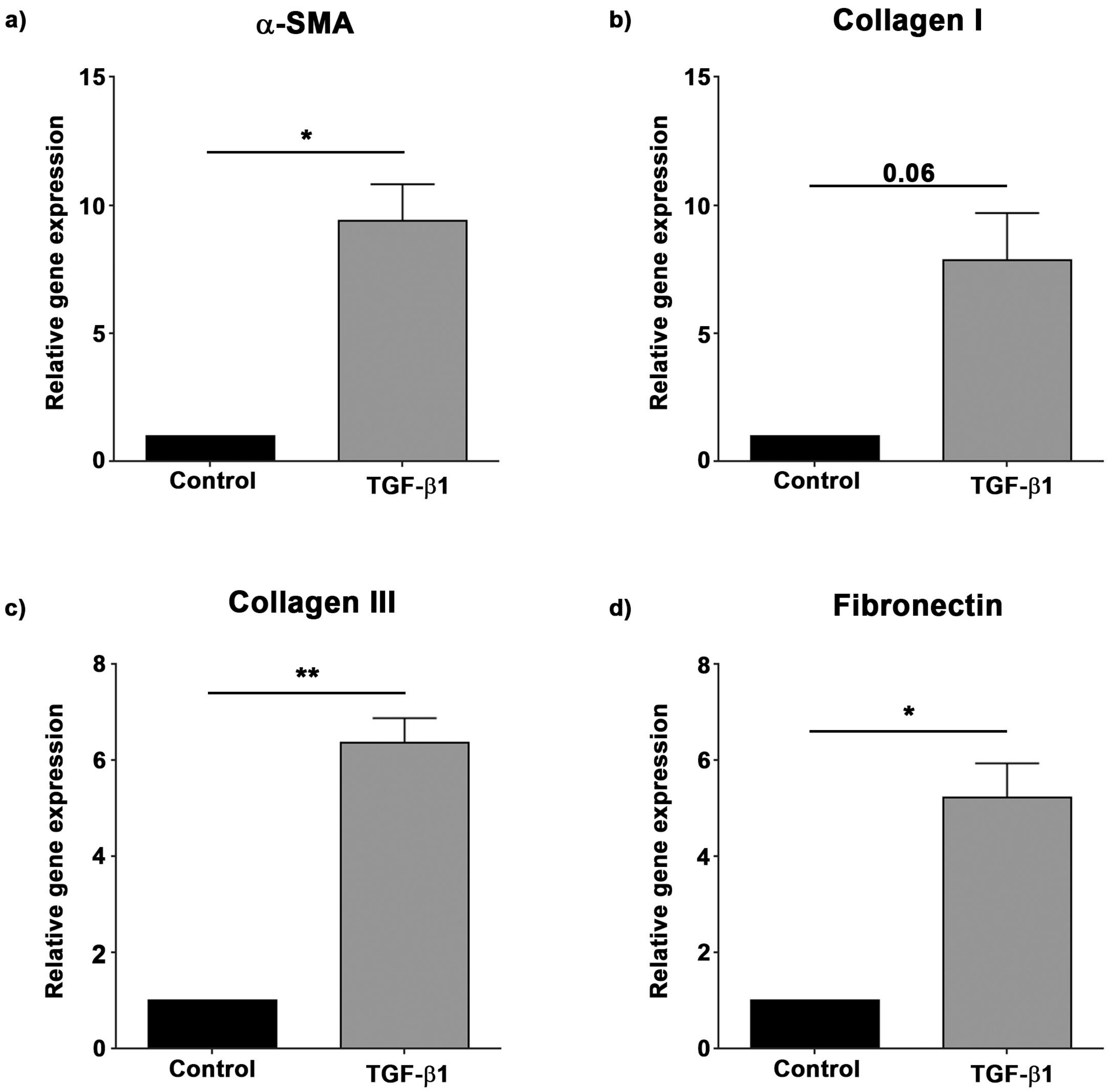
a)
α-SMA
15
10
5
0
*
Relative gene expression
Control
TGF-β1
b)
Collagen I
15
10
5
0
0.06
Relative gene expression
Control
TGF-β1
c)
Collagen III
8
6
4
2
0
**
Relative gene expression
Control
TGF-β1
d)
Fibronectin
8
6
4
2
0
*
Relative gene expression
Control
TGF-β1

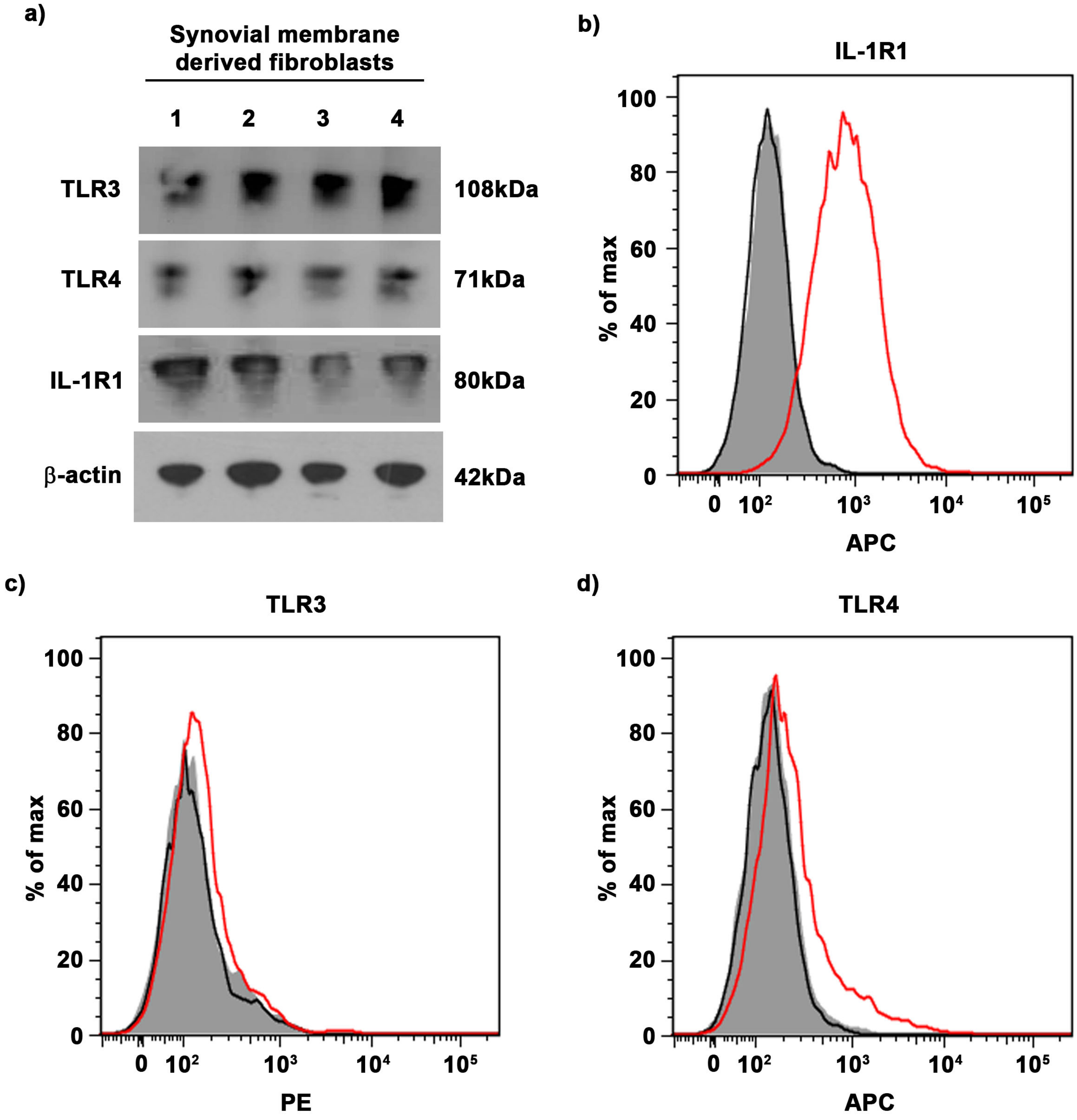
a)
Synovial membrane derived fibroblasts
1
2
3
4
TLR3
108kDa
TLR4
71kDa
IL-1R1
80kDa
β-actin
42kDa
b)
IL-1R1
% of max
APC
c)
TLR3
% of max
PE
d)
TLR4
% of max
APC

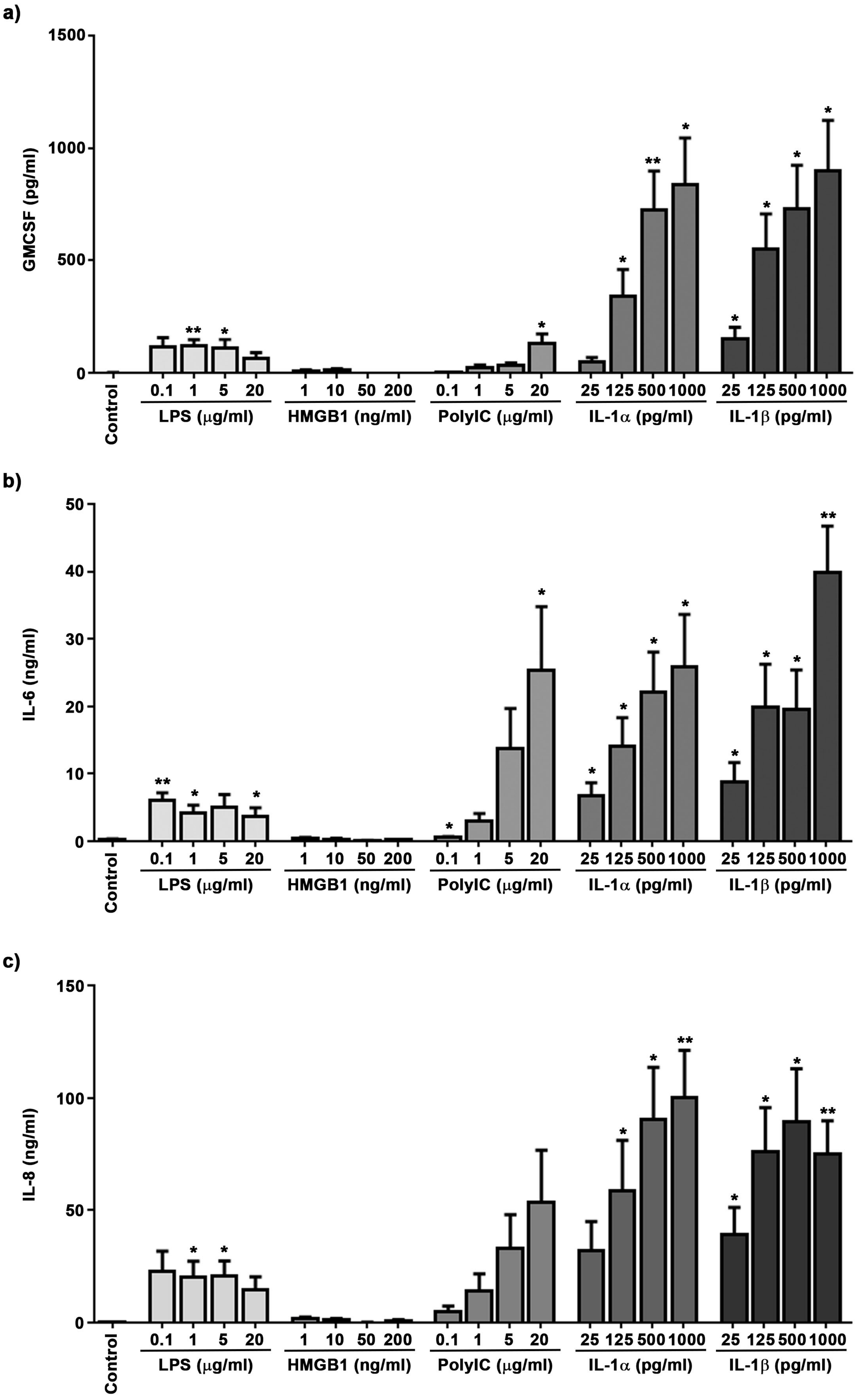
a)
GMCSF (pg/ml)
b)
IL-6 (ng/ml)
c)
IL-8 (ng/ml)
Control
0.1 1 5 20
LPS (μg/ml)
1 10 50 200
HMGB1 (ng/ml)
0.1 1 5 20
PolyIC (μg/ml)
25 125 500 1000
IL-1α (pg/ml)
25 125 500 1000
IL-1β (pg/ml)

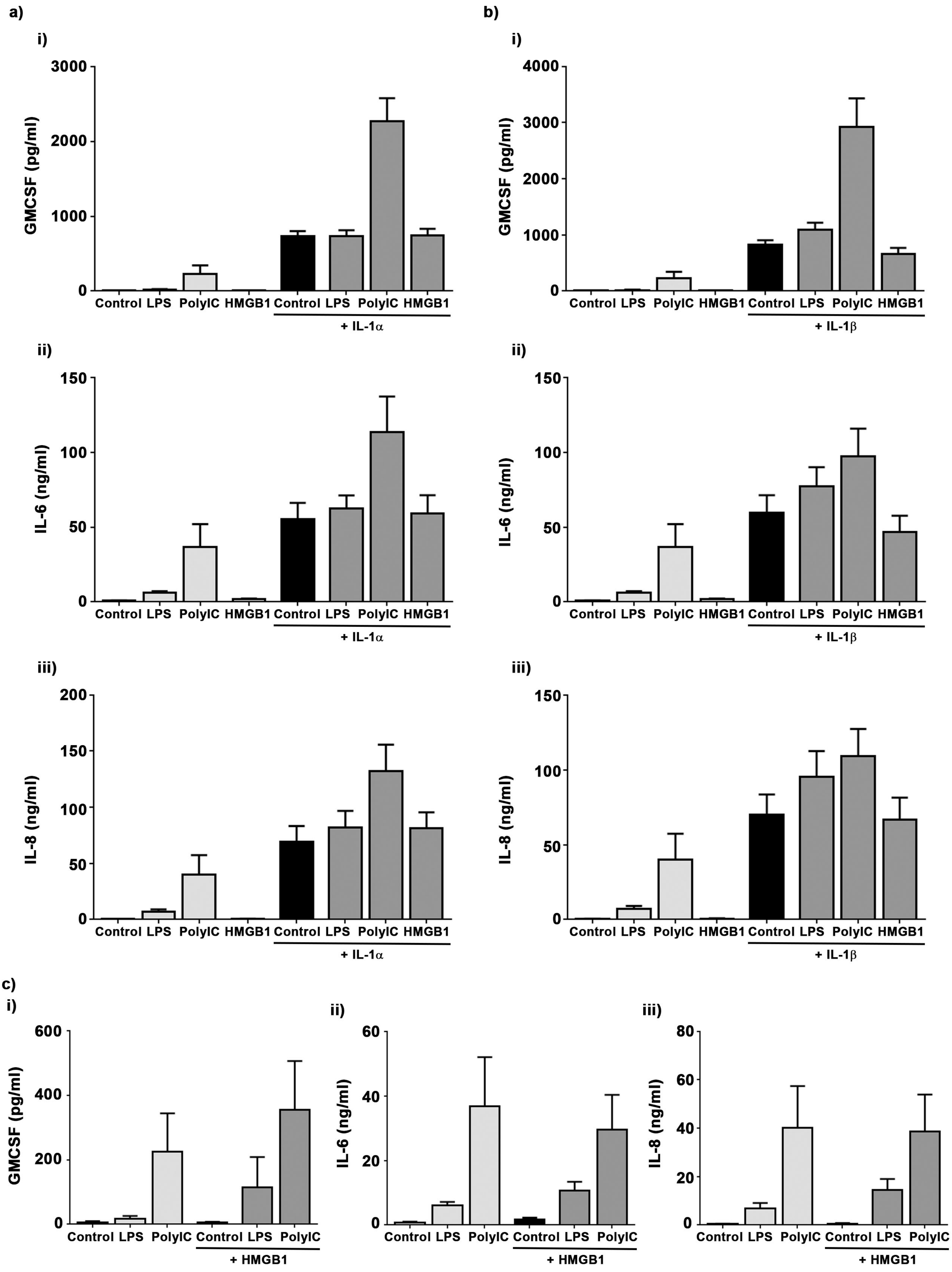
a)
i)
GMCSF (pg/ml)
0
1000
2000
3000
Control LPS PolyIC HMGB1 Control LPS PolyIC HMGB1
+ IL-1α
ii)
IL-6 (ng/ml)
0
50
100
150
Control LPS PolyIC HMGB1 Control LPS PolyIC HMGB1
+ IL-1α
iii)
IL-8 (ng/ml)
0
50
100
150
200
Control LPS PolyIC HMGB1 Control LPS PolyIC HMGB1
+ IL-1α
b)
i)
GMCSF (pg/ml)
0
1000
2000
3000
4000
Control LPS PolyIC HMGB1 Control LPS PolyIC HMGB1
+ IL-1β
ii)
IL-6 (ng/ml)
0
50
100
150
Control LPS PolyIC HMGB1 Control LPS PolyIC HMGB1
+ IL-1β
iii)
IL-8 (ng/ml)
0
50
100
150
Control LPS PolyIC HMGB1 Control LPS PolyIC HMGB1
+ IL-1β
c)
i)
GMCSF (pg/ml)
0
200
400
600
Control LPS PolyIC Control LPS PolyIC
+ HMGB1
ii)
IL-6 (ng/ml)
0
20
40
60
Control LPS PolyIC Control LPS PolyIC
+ HMGB1
iii)
IL-8 (ng/ml)
0
20
40
60
80
Control LPS PolyIC Control LPS PolyIC
+ HMGB1

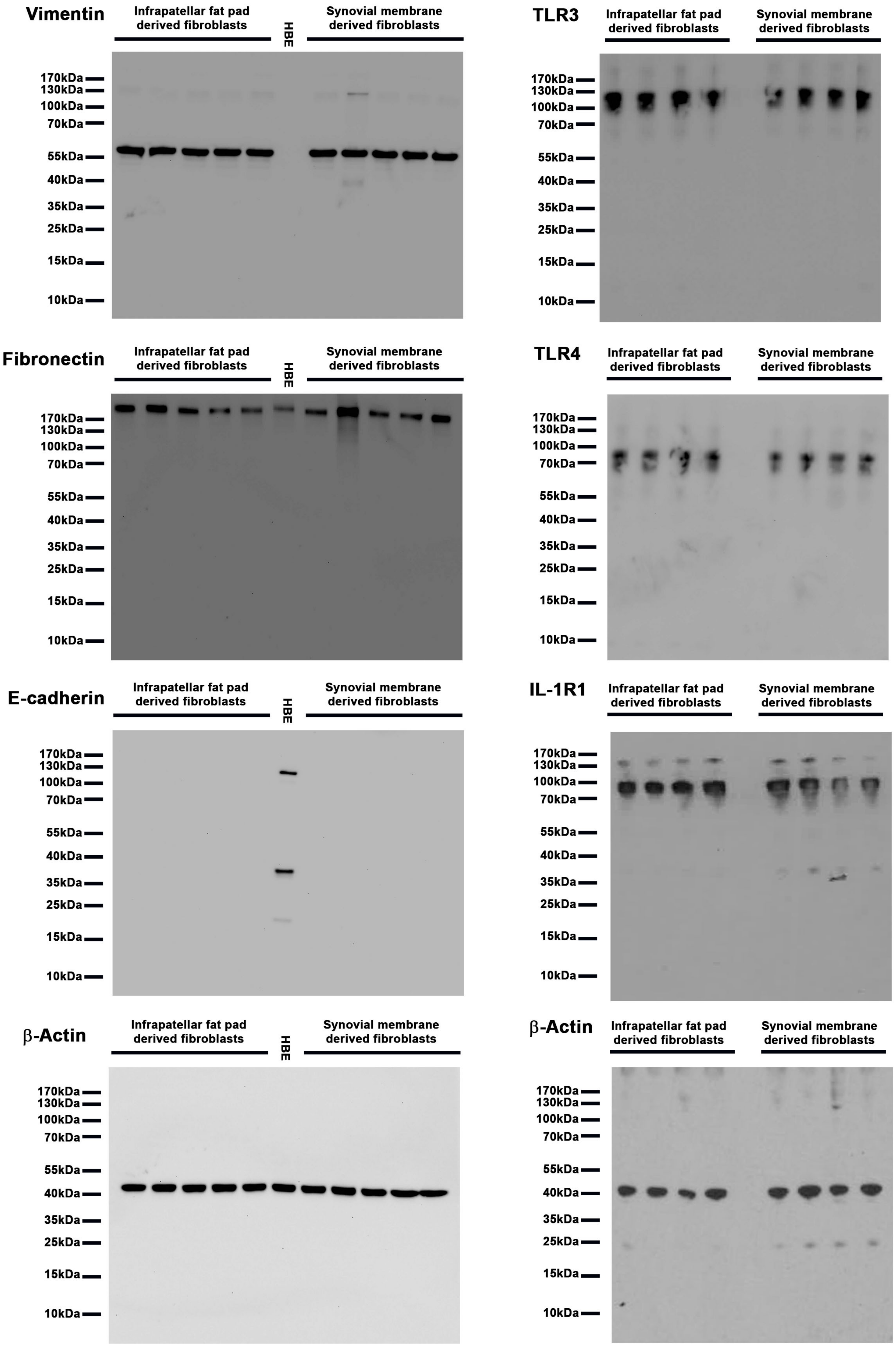
Vimentin
Infrapatellar fat pad derived fibroblasts
HBE
Synovial membrane derived fibroblasts
TLR3
Fibronectin
TLR4
E-cadherin
IL-1R1
β-Actin
170kDa
130kDa
100kDa
70kDa
55kDa
40kDa
35kDa
25kDa
15kDa
10kDa
